# SuperPATH—Current Status of Evidence and Further Investigations: A Scoping Review and Quality Assessment

**DOI:** 10.3390/jcm12165395

**Published:** 2023-08-19

**Authors:** Nikolai Ramadanov

**Affiliations:** Center of Orthopaedics and Traumatology, Brandenburg Medical School, University Hospital Brandenburg an der Havel, 14770 Brandenburg an der Havel, Germany; nikolai.ramadanov@gmail.com; Tel.: +49-177-740-66-33

**Keywords:** SuperPATH, total hip arthroplasty, hip replacement, surgical approach, scoping review

## Abstract

Background: SuperPATH is a novel minimally invasive technique for hip replacement that is gaining increasing attention. The aim of this review was to determine the nature, extent, and quality of current research evidence on SuperPATH and to identify areas for further investigations. Methods: A bibliometric search was conducted in PubMed up to 1 August 2023 using the search term “SuperPATH”. Data extraction and quality assessment were performed for relevant articles. Results: The bibliometric search yielded 51 articles on SuperPATH, 9 of which were meta-analyses, 11 were randomized controlled trials (RCTs), 4 were prospective non-RCTs, 12 were retrospective comparative studies, 11 were case series, and 4 were other article types. Most articles were published between 2015 and 2023, with a steady increase in publications per year. The articles originated from 13 countries, of which China was the most productive (35%). The quality assessment of the meta-analyses showed that 22.2% were of moderate quality, 66.7% were of low quality, and 11.1% were of critically low quality. The quality assessment of the RCTs showed that 36.4% had a low risk of bias (RoB), 27.2% revealed some concerns, and 36.4% had a high RoB. All studies were evaluated for content and taken into account in the formulation of recommendations and conclusions. Conclusions: The SuperPATH evidence varies from low to high quality. There is a steady increase in SuperPATH publications in the English-language literature and an uneven distribution of the article origins, with most articles coming from China. Consistent terminology should be used in the future, referring to the surgical approach as the direct superior approach (DSA) and to the surgical technique as SuperPATH. This review provides further concrete suggestions for future investigations and recommendations to improve study quality.

## 1. Introduction

**Figure 1:**
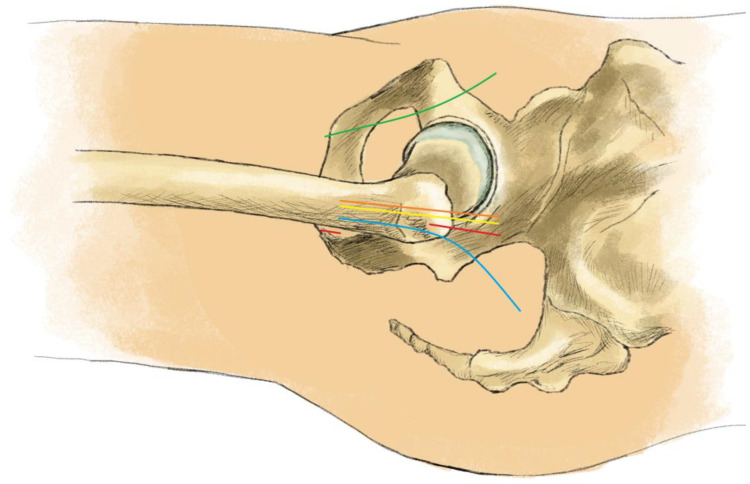
Topographical overview of the skin incision of the different hip approaches. Green line: direct anterior approach (DAA); orange line: anterolateral approach (ALA); yellow line: lateral approach (LA); red lines: direct superior approach (DSA) with additional stab incision; blue line: posterior/posterolateral approach (PA/PLA).

Total hip arthroplasty (THA) is one of the most successful surgical procedures of the 20th century [1]. It allows patients with various hip disorders to restore their joint function and improve their quality of life. In addition, THA or hemiarthroplasty (HA) is a surgical solution for the treatment of femoral neck fractures (FNFs) [2]. In an effort to improve the short-term outcomes after THA, several minimally invasive (MI) approaches have been invented and established in practice. In general, MI approaches are modifications of conventional approaches (CAs) that use smaller incisions and cause less soft tissue and muscle damage. Therefore, it is widely agreed that a hip approach can be considered MI if it meets two conditions: a skin incision length of <10 cm and, more importantly, no dissection of tendons and muscles. Three approaches fulfill these requirements: the direct anterior approach (DAA) using the anterior minimally invasive surgery (AMIS) surgical technique [3], the anterolateral approach (ALA) using the anterolateral minimally invasive (ALMI) surgical technique [4], and the direct superior approach (DSA), using the supercapsular percutaneously assisted total hip (SuperPATH) surgical technique [5]. Figure 1 provides a topographical overview of the skin incision of the different hip approaches.

SuperPATH was introduced in 2011 by James Chow [5], combining the advantages of two microposterior approaches—the supercapsular approach (SuperCap), developed by Stephen Murphy in 2004 [6], and the percutaneously assisted total hip approach (PATH), developed by Brad Penenberg in 2008 [7]. SuperCap allows access to the superior capsule using a DSA without causing relevant soft tissue and muscle damage [6]. PATH is a portal-assisted approach that provides the ability to achieve consistent and accurate acetabular cup positioning without causing relevant soft tissue and muscle damage [7]. Since its inception, SuperPATH has gained increasing support among orthopedic surgeons who have experienced its benefits in practice. However, it is striking that despite the promising results of SuperPATH, there are relatively few studies in the English language literature.

The aim of this study was to conduct a scoping review of the literature on SuperPATH to determine the nature, extent, and quality of current research evidence and to identify areas for further investigations.

## 2. Materials and Methods

### 2.1. Scoping Review

According to Grant and Booth [8], a scoping review is a “preliminary assessment of potential size and scope of the available research literature” that “aims to identify nature and extent of research evidence” [8]. According to Munn et al. [9], a scoping review answers broader questions than the widely known systematic review “beyond those related to the effectiveness of treatments or interventions” [9].

### 2.2. Search Strategy

A bibliometric search was performed in PubMed up to 1 August 2023 without language or year of publication restrictions. The exact search string used was “SuperPATH” with no filters applied. As the PubMed bibliometric search was intended to identify all published articles on SuperPATH, the selection criteria for article inclusion were as follows: any type of record in PubMed dealing with the topic of “SuperPATH”. Records were only excluded from the scoping review if they were not related to SuperPATH. This scoping review was conducted according to the Preferred Reporting Items for Systematic Reviews and Meta-Analyses Extension for Scoping Reviews (PRISMA-ScR) guidelines [10]. The PRISMA-ScR checklist [10] is available in the Supplementary Materials. The study protocol has been registered in the Open Science Framework, available online at: https://osf.io/nd2s5/ accessed on 25 June 2023.

### 2.3. Data Extraction and Quality Assessment

The following data were extracted from relevant articles: author’s name, year of publication, origin, and language of the article, article methods, main findings, and relevant additional information. In addition, the quality of relevant articles was assessed using the revised Measurement Tool to Assess Systematic Reviews (AMSTAR 2) [11] for meta-analyses, the revised Cochrane Risk of bias tool (RoB 2) [12] for randomized controlled trials (RCTs) and the Risk of bias in non-randomized studies of interventions (ROBINS-I) tool [13] for non-RCTs. The bibliometric search, data extraction, and quality assessment were conducted by two reviewers (NR, PMK). The inter-reviewer agreement was measured with the Cohen’s Kappa coefficient (κ). In the case of disagreement, consensus was reached after scientific discussion. 

## 3. Results

### 3.1. Search Results

The initial search returned 55 records [5,14,15,16,17,18,19,20,21,22,23,24,25,26,27,28,29,30,31,32,33,34,35,36,37,38,39,40,41,42,43,44,45,46,47,48,49,50,51,52,53,54,55,56,57,58,59,60,61,62,63,64,65,66,67] for further consideration. After full-text screening, 4 records [14,15,16,17] that used the term “SuperPath” with a different meaning were excluded. The bibliometric search with a complete inter-reviewer agreement (κ = 1.0) yielded 51 articles on SuperPATH [5,18,19,20,21,22,23,24,25,26,27,28,29,30,31,32,33,34,35,36,37,38,39,40,41,42,43,44,45,46,47,48,49,50,51,52,53,54,55,56,57,58,59,60,61,62,63,64,65,66,67], all of which were included in the scoping review. Of those 51 articles, 9 were meta-analyses [18,19,20,21,22,23,24,25,26], 11 were RCTs [27,28,29,30,31,32,33,34,35,36,37], 4 were prospective non-RCTs [38,39,40,41], 12 were retrospective comparative studies [42,43,44,45,46,47,48,49,50,51,52,53], 11 were case series [5,54,55,56,57,58,59,60,61,62,63], one was a bibliometric review [64], 2 were expert comments [65,66], and one was a study protocol [67].

**Figure 2:**
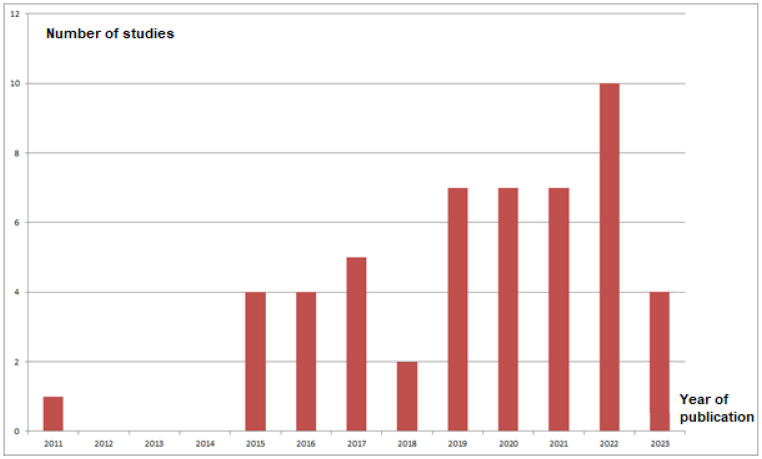
Temporal analysis of publication output.

**Figure 3:**
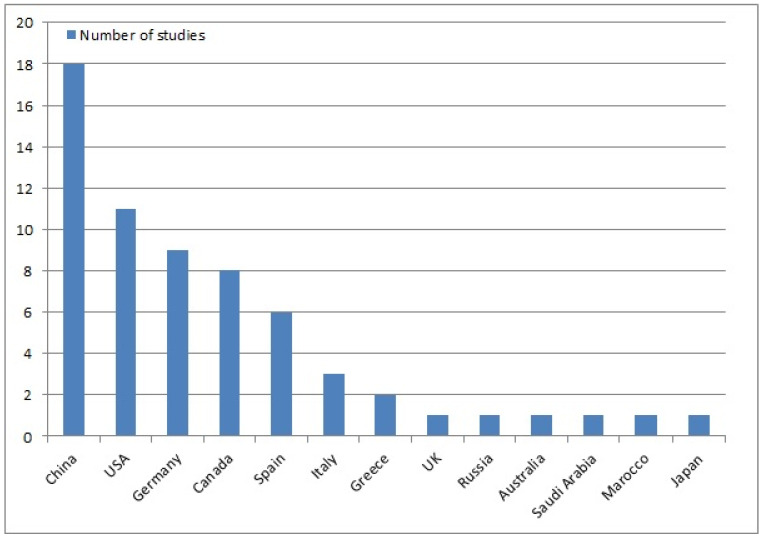
Publication output per country. Note that some studies originate from two countries, which is why the total number of studies is 63. USA: United States of America; UK: United Kingdom.

With the exception of the 2011 article [5] in which James Chow introduced SuperPATH, all other articles [18,19,20,21,22,23,24,25,26,27,28,29,30,31,32,33,34,35,36,37,38,39,40,41,42,43,44,45,46,47,48,49,50,51,52,53,54,55,56,57,58,59,60,61,62,63,64,65,66,67] were published between 2015 and 2023 (Figure 2). In 2015, 4 out of 51 articles (8%) were published on SuperPATH [42,54,55,67], while in 2022, 10 out of 51 articles (20%) were published on SuperPATH [22,23,24,25,35,50,51,52,53,62]. Figure 2 shows that there was a steady increase in the number of publications on SuperPATH per year. A total of 13 different countries were listed in the publication output (Figure 3). China was the most productive country, with 18 out of 51 articles (35%), followed by the United States with 11 articles (22%), Germany with 9 articles (18%), Canada with 8 articles (16%), and Spain with 6 articles (12%). 

### 3.2. Meta-Analyses on SuperPATH

The meta-analyses on SuperPATH [18,19,20,21,22,23,24,25,26] were published between 2020 and 2023. Of these 9 meta-analyses 5 (55.6%) were conventional meta-analyses [18,20,22,23,24], and 4 (44.4%) were network meta-analyses [19,21,25,26]. In 6 (66.7%) meta-analyses, the examined treatment was THA [19,21,22,23,25,26]. Two (22.2%) meta-analyses did not differentiate between THA and HA [20,24]. The first English language meta-analysis on SuperPATH included THA and HA [18]. It was the only meta-analysis to consider the influence of SuperPATH HA in a subgroup analysis [18]. Unfortunately, detailed data of the subgroup analysis performed were not reported [18]. Six (66.7%) [18,20,21,22,23,24] out of 9 meta-analyses compared the SuperPATH experimental group with a control group ofCAs without distinguishing between the individual approaches within the CA group. Four (44.4%) [19,21,25,26] out of 9 meta-analyses indirectly compared SuperPATH with DAA. In 2022, the first meta-analysis [25] comparing SuperPATH with the posterior approach (PA) was published. More recently, in 2023, the meta-analysis [26], with the largest sample size of 4859 patients, was published. SuperPATH was compared with the 2-incision approach, DAA, lateral approach (LA), MI LA, MI ALA, PA, and MI PA [26]. Seven (77.7%) of the meta-analyses included patients with any surgical indication for hip replacement [18,19,20,21,23,25,26]. Two (22.2%) out of 9 meta-analyses included either only patients with FNFs [22] or only osteoarthritis (OA) [24]. Six (66.7%) [18,19,21,23,25,26] out of 9 meta-analyses were limited to RCTs. Two (22.2%) out of 9 meta-analyses included RCTs and non-RCTs [20,24], and one (11.1%) out of 9 meta-analyses included only non-RCTs [22]. The following outcome parameters were reported: operation time [18,19,20,21,22,23,24,25,26], incision length [18,19,20,21,22,23,24,25], blood loss [18,19,20,21,22,23,24,25,26], pain visual analog scale (VAS) [18,19,20,21,22,23,24,26], Harris Hip Score (HHS) [18,19,20,21,22,23,24,25,26], acetabular cup positioning [18,19,20,21,23,24,25,26], length of hospital stay [18,20,22,24,26], complications [18,22,23,24,26], and quality of life [26]. Further details of the meta-analyses reviewed are provided in Table 1.

### 3.3. RCTs on SuperPATH

The 11 RCTs on SuperPATH [27,28,29,30,31,32,33,34,35,36,37] were published between 2017 and 2023. Of these 11 RCTs, 9 (81.8%) RCTs originated from China [27,28,29,30,31,32,33,34,36]. In 10 (90.9%) out of 11 RCTs, the treatment studied was THA [27,28,29,30,32,33,34,35,36,37]. One (9.1%) out of 11 RCTs investigated HAs [31]. Four (36.4%) [29,30,32,34] out of 11 RCTs compared SuperPATH with posterolateral approach (PLA), three (27.3%) [28,31,35] out of 11 RCTs compared SuperPATH with PA, two (18.2%) [27,36] out of 11 RCTs compared SuperPATH with LA, one (9.1%) [33] out of 11 RCTs compared SuperPATH with mini-incision PLA, and one (9.1%) [37] out of 11 RCTs compared SuperPATH with mini-incision PA. Four (36.4%) [27,29,30,34] out of 11 RCTs included patients with different surgical indications. Four (36.4%) [28,33,35,37] out of 11 RCTs included only patients with OA. Two (18.2%) [31,36] out of 11 RCTs included only patients with FNFs. One (9.1%) [32] out of 11 RCTs included only patients with avascular necrosis of the femoral head (ANFH). The sample size of the RCTs varied from 4 to 154 patients [27,32]. The following outcome parameters were reported: operation time [27,28,29,30,31,32,33,34,35,36,37], incision length [27,28,29,30,31,32,33,34,36], blood loss [27,28,29,30,31,32,33,34,35,36,37], pain VAS [27,28,30,31,32,33,34,35,36,37], functional outcome by HHS [27,28,29,30,31,32,33,34,36,37], by Oxford Hip Score (OHS) [35], and by Hip disability and osteoarthritis outcome score (HOOS) [37], acetabular cup positioning [27,28,30,32,33,34,35,36,37], length of hospital stay [27,28,30,32,33,34,35,36,37], complications [27,28,30,32,33,34,35,36,37], Short Form 36 (SF-36) [29], laboratory parameters [30,32,33,34,35,36,37], Barthel Index [31], and range of motion (ROM) [30,32,33,37]. Further details of the RCTs reviewed are provided in Table 2. 

### 3.4. Prospective Non-RCTs on SuperPATH

The 4 prospective non-RCTs on SuperPATH [38,39,40,41] were published between 2017 and 2020. Every prospective non-RCT originated from a different country [38,39,40,41]. All prospective non-RCTs examined THA [38,39,40,41]. One (25%) [40] out of 4 prospective non-RCTs compared SuperPATH with PLA, one (25%) [39] out of 4 prospective non-RCTs compared SuperPATH with PA, one (25%) [41] out of 4 RCTs compared SuperPATH with LA, one (25%) [38] out of 4 prospective non-RCTs compared the same SuperPATH group at different time points with itself. Two (50%) [38,41] out of 4 prospective non-RCTs included patients with different surgical indications. One (25%) [39] out of 4 prospective non-RCTs included patients with OA. One (25%) [40] out of 4 prospective non-RCTs included patients with FNFs. The sample size of the RCTs varied from 48 to 110 patients [40,41]. The following outcome parameters were reported: operation time [39,40,41], incision length [40,41], blood loss [39,40,41], pain VAS [40,41], functional outcome by HHS [39,40,41], by Western Ontario and MacMaster Universities Osteoarthritis Index (WOMAC) [39], acetabular cup positioning [39,41], length of hospital stay [39,40,41], complications [40,41], SF-36 [39], laboratory parameters [41], and brake reaction time [38]. Further details of the prospective non-RCTs reviewed are provided in Table 2.

### 3.5. Quality Assessment

The quality assessment using AMSTAR 2 [11] with the high inter-reviewer agreement (κ = 0.98) showed that 2 (22.2%) [23,26] out of 9 meta-analyses were of moderate quality level, while 6 (66.7%) were of low-quality level [18,19,20,21,22,25], and one (11.1%) was of critically low-quality level [24]. The quality assessment according to each critical domain in AMSTAR 2 is provided in Table 3.

The quality assessment using the Cochrane RoB 2 tool [12] with high inter-reviewer agreement (κ = 0.98) showed that 4 out of 11 RCTs (36.4%) had a low RoB [32,33,34,37], while 3 (27.2%) showed some concerns [29,30,35], and 4 (36.4%) had a high RoB [27,28,31,36]. The quality assessment according to each item in Cochrane RoB 2 tool [12] is provided in Table 4.

The quality assessment using ROBINS-I tool [13] with a complete inter-reviewer agreement (κ = 1.00) showed that one (25%) out of 4 prospective non-RCTs had a low RoB [38], while one (25%) had a moderate RoB [40], and 2 (50%) had a serious RoB [39,41]. The quality assessment according to each item in ROBINS-I tool [13] is provided in Table 5.

## 4. Discussion

The scoping review in PubMed revealed that as of 1 August 2023, only 51 articles on SuperPATH had been published in the English language specialist literature. Of these, 9 were meta-analyses [18,19,20,21,22,23,24,25,26], 11 were RCTs [27,28,29,30,31,32,33,34,35,36,37], 4 were prospective non-RCTs [38,39,40,41], 12 were retrospective comparative studies [42,43,44,45,46,47,48,49,50,51,52,53], 11 were case series [5,54,55,56,57,58,59,60,61,62,63], and 4 were other types of articles [64,65,66,67]. The quality assessment of the articles showed a relatively even distribution from a low to high level of quality. Looking at the frequency of publications over the last few years, there has been a steady increase reflecting the growing interest in the SuperPATH hip replacement technique. Analysis of the origin of each article showed that a large proportion came from China. Furthermore, a quick search of the English version of the Chinese scientific database “China National Knowledge Infrastructure” (CNKI) shows that there are significantly more published articles on SuperPATH, including high-quality RCTs, than in the Western world. One can speculate about this unequal distribution of the origin of the articles. The MicroPort company, which produces implants for MI hip arthroplasty (including SuperPATH), is headquartered in Shanghai (China). It is, therefore, questionable whether the establishment of SuperPATH is being specifically promoted by Chinese authors or whether articles on SuperPATH are more likely to be rejected by Western journals. Regardless of the reason, this uneven distribution suggests a relevant publication bias.

An analysis of the 51 articles included in the review reveals inconsistencies in the use of terminology related to SuperPATH. Lack of standardization of terminology is not uncommon when innovations are introduced into the literature. Most authors refer to SuperPATH as an approach to the hip joint. However, SuperPATH is not a surgical approach. It is a novel surgical technique that uses a DSA to the hip joint [65]. The DSA has evolved from the posterior and, specifically, from microposterior approaches. For this reason, some authors classify it as a PA. This understanding also needs to be corrected, as the names of the hip approaches are based on their anatomical relationship to the greater trochanter (Figure 1). Therefore, the scientific community should agree to refer to the approach as DSA and to the surgical technique as SuperPATH in the future.

A detailed analysis of the articles published on SuperPATH showed that information on the use of bone cement was very rarely reported. Only 3 [28,35,37] out of 11 RCTs reported that they did not use bone cement. In the other cases, it can be assumed that cementless implants were chosen, but this cannot be said with certainty because the SuperPATH implants also have cemented variants. The use of bone cement is not uncommon, especially in FNFs. This lack of information is a major limitation of many publications, which is consequently reflected in the synthesis of the articles in meta-analyses [18,19,20,21,22,23,24,25,26]. Furthermore, it is not clear whether the RCTs included the additional stab incision in the total incision length of SuperPATH or whether only the main incision was reported. Logically, this limitation also appears in the meta-analyses [18,19,20,21,22,23,24,25,26]. Furthermore, the additional stab incision in HA using the SuperPATH technique can be omitted [57], as the additional portal is only required to ream the acetabular cup. However, future studies should explicitly clarify whether the additional stab incision was omitted in SuperPATH HA. No matter how small this additional stab incision may be, its omission still suggests, to some extent, less soft tissue and muscle damage. Another important piece of information that was rarely reported is the blinding process in RCTs. This shortcoming consequently leads to a lower level of quality in the RoB assessment [27,28,29,30,31,35,36].

The next interesting point raised by 4 articles of this scoping review [39,42,47,55] is the learning curve of the SuperPATH technique. In an analysis of 50 consecutive SuperPATH operations performed by a non-developing surgeon, the learning curve was assessed using the operation time as a surrogate [42]. Rasuli and Gofton found that the mean operation time of SuperPATH was 101.7 ± 18.3 min, with a further decrease after case 50 [42]. In their analysis of 78 consecutive SuperPATH operations (80 hips) performed by the same surgeon, the authors Lei et al. came to similar findings [47]. In addition, they concluded that surgeons who are familiar with the conventional PLA may achieve greater comfort with SuperPATH after 40 cases of surgery [47]. In a prospective study, Más Martínez et al. compared 30 cases of SuperPATH THA with 60 cases of conventional PA THA [39]. The learning curve of SuperPATH provided similar outcomes to the conventional PA within the first year after surgery [39]. A case series of 100 consecutive patients by Della Torre et al. [55] analyzed the SuperPATH THA component position and seating, femoral offset, and leg length. The authors concluded that the implant position was optimal within the learning curve for the described THA safe zones [55]. The learning curve described in SuperPATH can certainly be explained by the fact that SuperPATH is a novel technique that requires special instruments and surgical skills. The interesting question here is how the cost of THAs compares. Obviously, there is a cost associated with the purchase of the specialized SuperPATH instruments. Whether these costs can be offset by the better short-term outcome of SuperPATH [18,19,20,21,22,23,24,25] and the associated shorter hospital stay [25] remains to be scientifically investigated.

Based on the review of the articles, the following suggestions for future research emerge: (1) There are only two meta-analyses comparing the individual CA separately with SuperPATH [25,26]. Most meta-analyses grouped the CAs together [18,20,21,22,23,24], which is a serious limitation. Further publications are needed to overcome this limitation. (2) SuperPATH has already been compared with DAA in meta-analyses [19,21,25,26]. However, this could only be performed via an indirect comparison using a network meta-analysis, as there are still no published RCTs directly comparing SuperPATH with DAA. These preliminary indirect comparisons showed overall better results for SuperPATH. The validity of these preliminary results urgently needs to be confirmed by direct comparisons in RCTs and meta-analyses. (3) There are no meta-analyses comparing SuperPATH with other MI techniques (other than DAA) or to robotic-assisted THA techniques. (4) Although there are some RCTs [30,32,33,34,35,36,37] that have evaluated the laboratory parameters after SuperPATH hip replacement, these data have not yet been pooled in a meta-analysis. A meta-analysis of laboratory parameters would provide a reliable picture of the extent of tissue damage and blood loss. (5) Furthermore, there is no meta-analysis examining the outcome of SuperPATH HA. Research on this topic is important because, as mentioned above, there are key differences in surgical approach and technique between SuperPATH THA and SuperPATH HA. The impact of omitting the additional stab incision in HA needs to be investigated. (6) While many meta-analyses [18,19,20,21,23,24,25,26] and RCTs [27,28,30,32,33,34,35,36,37] have investigated the acetabular cup positioning of SuperPATH, there is no radiographic analysis of the stem positioning in the literature. Filling this scientific gap is important because the SuperPATH technique involves implanting the femoral stem in situ prior to femoral neck resection. This is a completely new procedure in arthroplasty, the results of which need to be studied. (7) There is also a lack of studies in the literature on revision THA through SuperPATH and on mid- and long-term outcomes of SuperPATH.

After reviewing the results of the 9 meta-analyses [18,19,20,21,22,23,24,25,26] and 11 RCTs [27,28,29,30,31,32,33,34,35,36,37], the current state of the literature allows for the final conclusion that SuperPATH showed better short-term THA outcomes than the CA group [18,20,21,22,23,24,27,28,29,30,31,32,34,35,36]. This improvement relates to important outcome parameters such as blood loss, pain score, and functional outcome, while acetabular cup positioning angles and complication rates remained comparable. In addition, preliminary results of indirect comparisons show a better short-term outcome of SuperPATH THA compared with DAA THA [19,21,25,26].

To be further investigated:

- RCTs and meta-analyses of SuperPATH vs. each CA separately (ALA, LA, PA, PLA);
- RCTs and meta-analyses of SuperPATH vs. DAA in direct comparison;
- RCTs and meta-analyses of SuperPATH vs. other MI techniques (except of DAA) or vs. robotic-assisted techniques;
- Meta-analyses of the laboratory parameters of SuperPATH vs. other techniques;
- RCTs and meta-analyses of SuperPATH HA;
- RCTs and meta-analyses of the stem positioning through SuperPATH;
- RCT and meta-analyses of revision THA through SuperPATH;
- RCTs and meta-analyses of mid- and long-term outcomes of SuperPATH.

Recommendation for future studies:

- Standardization of terminology: SuperPATH is the designation of a hip replacement technique that uses a direct superior approach (DSA);
- Report the use of bone cement;
- Report information on the additional stab incision:
- Do you report an added incision length of both incisions (additional stab incision + main incision) in SuperPATH THA or just the main incision?
- Did you omit the additional stab incision in SuperPATH HA?

The following limitations of this scoping review should be noted: (1) The bibliometric search was limited to only one database (PubMed). (2) The meta-analyses and RCTs on SuperPATH were given more attention in this scoping review because they are at the forefront of evidence-based medicine. However, there may be relevant findings in the other studies. (3) The recommendations and conclusions of this scoping review are based on a scientific interpretation of the primary studies, but there may still be some degree of subjectivity based on the author’s expert opinion.

## 5. Conclusions

The research evidence on SuperPATH varies from low to high quality. There is a steady increase in publications on SuperPATH in the English language literature and an uneven distribution of article origins, with most articles coming from China. The current state of the literature suggests that SuperPATH THA has a better short-term outcome than CA THA. Consistent terminology should be used in the future, referring to the surgical approach as DSA and to the surgical technique as SuperPATH. Numerous other gaps in the specialist literature were identified. This review provides concrete suggestions for future investigations and recommendations to improve study quality.

## Figures and Tables

**Table 1:** Characteristics of meta-analyses on SuperPATH. ISSN: International Standard Serial Number; THA: total hip arthroplasty; HA: hemiarthroplasty; CAs: conventional approaches; OA: osteoarthritis; ANFH: avascular necrosis of the femoral head; FNF: femoral neck fracture; CNKI: China National Knowledge Infrastructure; RCT: randomized controlled trial; RoB: risk of bias; LoE: Level of evidence; NOS: NewcastleOttawa Scale; PB: Publication bias; MINORS: Methodological index for non-randomized studies; DAA: direct anterior approach; PA: posterior approach; LA: lateral approach, MI: minimally invasive; ALA: anterolateral approach; 1: operation time; 2: incision length; 3: blood loss; 4: pain VAS: pain visual analog scale; 5: HHS: Harris Hip Score; 6: acetabular cup positioning; 7: length of hospital stay; 8: complications; 9: quality of life.

First Author	Year of Publication	Origin	Language	Journal (ISSN)	Study Design	Study Protocol	Operation	Compared with:	Surgical Indication	Databases Searched	Included Studies	Quality Assessment	Number of Patients	Outcome Parameters	Remarks
Ramadanov N et al. [18]	2020	Germany/ Spain	English	Journal of Orthopedic Surgery and Research (1749–799X)	Meta-analysis	Yes	THA, HA	CAs	OA, ANFH, FNF	PubMed, Cochrane Library, Clinical Trials, CNKI, Google Scholar	12 RCTs	RoB, LoE,	726	1, 2, 3, 4, 5, 6, 7, 8	First meta-analysis of SuperPATH in the English-language literature; HA subgroup analysis
Ramadanov N et al. [19]	2021	Germany/ Spain	English	Journal of Orthopedic Surgery and Research (1749–799X)	Network meta-analysis	Yes	THA	DAA	OA, ANFH, FNF	PubMed, Cochrane Library, Clinical Trials, CNKI, Google Scholar	16 RCTs	RoB, LoE	1392	1, 2, 3, 4, 5, 6	First meta-analysis of SuperPATH vs. DAA
Ge Y et al. [20]	2021	China	English	BioMed Research International (2314–6141)	Meta-analysis	Yes	THA, HA	CAs	OA, FNF	PubMed, Embase, Cochrane Library	3 RCTs, 3 non-RCTs	RoB, NOS	526	1, 2, 3, 4, 5, 6, 7	Limitation: both THA, HA
Ramadanov et al. [21]	2021	Germany/ Spain	English	Orthopedics and Traumatology: Surgery and Research (1877–0568)	Network meta-analysis	Yes	THA	DAA, CAs	OA, ANFH, FNF	PubMed, Scopus, Web of Science, Cochrane Library, Clinical Trials Cinahl, CNKI,	24 RCTs	RoB, LoE	2074	1, 2, 3, 4, 5, 6	Update of Ramadanov N et al. [19]
Zhao F et al. [22]	2022	China	English	Geriatric Orthopedic Surgery and Rehabilitation (2151–4593)	Meta-analysis	No	THA	CAs	FNF	PubMed, Embase, Scopus, Web of Science, Cochrane Library, CNKI, Wanfan	9 Non-RCTs	RoB, MINORS, PB	694	1, 2, 3, 4, 5, 7, 8	First meta-analysis of SuperPATH THA in FNFs
Ramadanov N [23]	2022	Germany	English	Orthopedic Surgery (1757–7861)	Meta-analysis	Yes	THA	CAs	OA, ANFH, FNF	PubMed, Cochrane Library, Clinical Trials, CNKI, Google Scholar	14 RCTs	RoB, LoE, PB	1021	1, 2, 3, 4, 5, 6, 8	Update of Ramadanov N et al. [18]
Joseph VM et al. [24]	2022	United Kingdom	English	HIP International (1724–6067)	Meta-analysis	No	THA, HA	CAs	OA	PubMed, Embase, Scopus, Web of Science, Cochrane Library, Cinahl EMCare	3 RCTs,4 non-RCTs	RoB	730	1, 2, 3, 4, 5, 6, 7, 8	Limitation: both THA, HA
Ramadanov N et al. [25]	2022	Germany/ Spain	English	Scientific Reports (2045–2322)	Network meta-analysis	Yes	THA	DAA, PA	OA, ANFH, FNF, Dysplasia	PubMed, Embase, Cochrane Library, Clinical trials, CNKI	20 RCTs	RoB, LoE	1501	1, 2, 3, 5, 6	First meta-analysis of SuperPATH vs. PA
Yan L et al. [26]	2023	China/ Canada	English	JAMA Network Open (2574–3805)	Network meta-analysis	Yes	THA	2-incision approach, DAA, LA, MI LA, MI ALA, PA, MI PA	Any indication	PubMed, Embase, Cochrane Library, Clinical Trials	63 RCTs	RoB, LoE, PB	4859	1, 3, 4, 5, 6, 7, 8, 9	Highest sample size

**Table 2:** Characteristics of RCTs and non-RCTs on SuperPATH. ISSN: International Standard Serial Number; THA: total hip arthroplasty; HA: hemiarthroplasty; RCT: randomized controlled trial; NR: not reported; LA: lateral approach; OA: osteoarthritis; ANFH: avascular necrosis of the femoral head; FNF: femoral neck fracture; PA: posterior approach; PLA: posterolateral approach, 1: operation time; 2: incision length; 3: blood loss; 4: pain VAS: pain visual analog scale; 5: functional outcome: HHS: Harris Hip Score, OHS: Oxford Hip Score, HOOS: Hip disability and osteoarthritis outcome score, WOMAC: Western Ontario and MacMaster Universities Osteoarthritis Index; 6: acetabular cup positioning; 7: length of hospital stay; 8: complications; 9: SF-36: Short Form 36; 10: laboratory parameters; 11: Barthel index; 12: ROM: range of motion.

First Author	Year of Publication	Origin	Language	Journal (ISSN)	Study Design	THA or HA	Cement	Compared with:	Surgical Indication	Number of Patients	Outcome Parameters	Remarks
Yan T et al. [27]	2017	China	Chinese/ English	Zhongguo Xiu Fu Chong Jian Wai Ke Za Zhi (1002–1892)	RCT	THA	NR	LA	OA, ANFH, FNF, Dysplasia	154	1, 2, 3, 4, 5 (HHS), 6, 7, 8	Uni- and bilateral THA
Xie J et al. [28]	2017	China	English	Journal of Orthopedic Surgery and Research (1749–799X)	RCT	THA	Cementless	PA	OA	92	1, 2, 3, 4, 5 (HHS), 6, 7, 8	No blinding
Yuan H et al. [29]	2018	China	Chinese/ English	Zhongguo Xiu Fu Chong Jian Wai Ke Za Zhi (1002–1892)	RCT	THA	NR	PLA	OA, ANFH, FNF, Dysplasia	84	1, 2, 3, 5 (HHS), 9	-
Ouyang C et al. [30]	2018	China	Chinese	Zhongguo Xiu Fu Chong Jian Wai Ke Za Zhi (1002–1892)	RCT	THA	NR	PLA	OA, ANFH	24	1, 2, 3, 4, 5 (HHS), 6, 7, 8, 10, 12	Low sample size
Jianbo J et al. [31]	2019	China	English	Injury (0020–1383)	RCT	HA	NR	PA	FNF	100	1, 2, 3, 4, 5 (HHS), 11	First RCT on SuperPATH HA in FNF; no blinding
Meng W et al. [32]	2019	China/ Germany	English	Musculoskeletal Disorders (1471–2474)	RCT	THA	NR	PLA	ANFH	4	1, 2, 3, 4, 5 (HHS), 6, 7, 8, 10, 12	Bilateral; very low sample size
Meng W et al. [33]	2021	China/ Germany	English	Annals of Translational Medicine (2305–5847)	RCT	THA	NR	Mini-incision PLA	OA	40	1, 2, 3, 4, 5 (HHS), 6, 7, 8, 10, 12	-
Li X et al. [34]	2021	China	English	Asian Journal of Surgery (1015–9584/)	RCT	THA	NR	PLA	ANFH, FNF	96	1, 2, 3, 4, 5 (HHS), 6, 7, 8, 10	-
Khoja YT et al. [35]	2022	Canada/ Saudi Arabia	English	Clinical Orthopedics and Related Research (0009–921X)	RCT	THA	Cementless	PA	OA	46	1, 3, 4, 5 (OHS), 6, 7, 8, 10	-
Shen J et al. [36]	2023	China	English	Journal of Orthopedic Surgery and Research (1749–799X)	RCT	THA	NR	LA	FNF	120	1, 2, 3, 4, 5 (HHS), 6, 7, 8, 10	No blinding
Korytkin AA et al. [37]	2023	Russia/ Marocco	English	HIP International (1724–6067)	RCT	THA	Cementless	Mini-incision PA	OA	49	1, 3, 4, 5 (HHS, HOOS), 6, 7, 8, 10, 12	-
Qurashi S et al. [38]	2017	Australia/ USA	English	Journal of Arthroplasty (0883–5403)	Prospective non-RCT	THA	Cementless	SuperPATH	OA, ANFH, Dysplasia	100	Brake reaction time after SuperPATH THA	-
Más Martínez J et al. [39]	2019	Spain	English/ Spanish	Revista Espanola de Cirugia Ortopedica y Traumatologia (1988–8856)	Prospective non-RCT	THA	Cementless	PA	OA	90	1, 3, 5 (HHS, WOMAC), 6, 7, 9	-
Wang XD et al. [40]	2020	China	English	Orthopedic Surgery (1757–7861)	Prospective non-RCT	THA	NR	PLA	FNF	110	1, 2, 3, 4, 5 (HHS), 7, 8	-
Tottas S et al. [41]	2020	Greece	English	Journal of Orthopedics (0972–978X)	Prospective non-RCT	THA	Cementless	LA	OA, ANFH, Dysplasia	48	1, 2, 3, 4, 5 (HHS), 6, 7, 8, 10	-

**Table 3:** Meta-analyses quality assessment, using AMSTAR 2 (A MeaSurement Tool to Assess Systematic Reviews 2). Possible assessment results: “Critically low”, “Low”, “Moderate”, “High”. Note that the labels refer to quality and not RoB, e.g., “low” is bad, and “high” is good. The meaning of “low” and “high” is reversed in the RoB 2 tool compared to AMSTAR 2. RoB: risk of bias.

Author	Protocol Registered before Commencement of the Review	Adequacy of the Literature Search	Justification for Excluding Individual Studies	RoB from Individual Studies Being Included in the Review	Appropriateness of Meta-Analytical Methods	Consideration of RoB When Interpreting the Results of the Review	Assessment of Presence and Likely Impact of Publication Bias	Overall Quality
Ramadanov N et al. [18]	High	High	Moderate	High	High	Moderate	Low	Low
Ramadanov N et al. [19]	High	High	Moderate	High	High	Moderate	Low	Low
Ge Y et al. [20]	High	High	High	High	High	Moderate	Low	Low
Ramadanov et al. [21]	High	High	Moderate	High	High	Moderate	Low	Low
Zhao F et al. [22]	Low	High	High	High	High	Moderate	High	Low
Ramadanov N [23]	High	High	Moderate	High	High	High	High	Moderate
Joseph VM et al. [24]	Low	Low	Moderate	High	High	Moderate	Low	Critically low
Ramadanov N et al. [25]	High	High	Moderate	High	High	Moderate	Low	Low
Yan L et al. [26]	High	Moderate	High	High	High	Moderate	High	Moderate

**Table 4:** RCT quality assessment using RoB 2 tool. Possible assessment results: “High RoB”, “Some concerns”, “Low RoB”. RCT: randomized controlled trial; RoB: risk of bias.

Author	Bias Arising from the Randomization Process	Bias Due to Deviation from Intended Interventions	Bias Due to Missing Outcome Data	Bias in Measurement of the Outcome	Bias in Selection of the Reported Result	Overall RoB
Yan T et al. [27]	Some concerns	High RoB	Low RoB	Low RoB	Low RoB	High RoB
Xie J et al. [28]	High RoB	Low RoB	Low RoB	Low RoB	Low RoB	High RoB
Yuan H et al. [29]	Some concerns	Low RoB	Low RoB	Low RoB	Low RoB	Some concerns
Ouyang C et al. [30]	Some concerns	Low RoB	Low RoB	Low RoB	Low RoB	Some concerns
Jianbo J et al. [31]	High RoB	Low RoB	Low RoB	Low RoB	Low RoB	High RoB
Meng W et al. [32]	Low RoB	Low RoB	Low RoB	Low RoB	Low RoB	Low RoB
Meng W et al. [33]	Low RoB	Low RoB	Low RoB	Low RoB	Low RoB	Low RoB
Li X et al. [34]	Low RoB	Low RoB	Low RoB	Low RoB	Low RoB	Low RoB
Khoja YT et al. [35]	Some concerns	Low RoB	Low RoB	Low RoB	Low RoB	Some concerns
Shen J et al. [36]	High RoB	Low RoB	Low RoB	Low RoB	Low RoB	High RoB
Korytkin AA et al. [37]	Low RoB	Low RoB	Low RoB	Low RoB	Low RoB	Low RoB

**Table 5:** Non-RCT quality assessment, using ROBINS-I (Risk of bias in non-randomized studies of interventions) tool. Possible assessment results: “Critical RoB”, “Serious RoB”, “Moderate RoB”, “Low RoB”. RCT: randomized controlled trial; RoB: risk of bias.

First Author	Bias Due to Confounding	Bias in Selection of Participants into the Study	Bias in Classification of Interventions	Bias Due to Deviations from Intended Interventions	Bias Due to Missing Data	Bias in Measurement of the Outcome	Bias in Selection of the Reported Result	Overall RoB
Qurashi S et al. [38]	Low RoB	Low RoB	Low RoB	Low RoB	Low RoB	Low RoB	Low RoB	Low RoB
Más Martínez J et al. [39]	Low RoB	Serious RoB	Low RoB	Low RoB	Low RoB	Moderate RoB	Low RoB	Serious RoB
Wang XD et al. [40]	Low RoB	Moderate RoB	Low RoB	Low RoB	Low RoB	Moderate RoB	Low RoB	Moderate RoB
Tottas S et al. [41]	Moderate RoB	Serious RoB	Low RoB	Low RoB	Low RoB	Low RoB	Low RoB	Serious RoB

## Data Availability

Not applicable.

## Supplementary Materials

The following supporting information can be downloaded at: https://www.mdpi.com/article/10.3390/jcm12165395/s1. Table S1: Preferred Reporting Items for Systematic reviews and Meta-Analyses extension for Scoping Reviews (PRISMA-ScR) Checklist. Reference [10] is cited in Supplementary Materials.

## Abbreviations

ALMI	anterolateral minimally invasive
ALA	anterolateral approach
AMIS	anterior minimally invasive surgery
AMSTAR	A MeaSurement Tool to Assess Systematic Reviews
ANFH	avascular necrosis of the femoral head
CA	conventional approach
CNKI	China National Knowledge Infrastructure
DAA	direct anterior approach
DSA	direct superior approach
FNF	femoral neck fracture
HA	hemiarthroplasty
HHS	Harris Hip Score
HOOS	Hip disability and osteoarthritis outcome score
LA	lateral approach
MI	minimally invasive
OA	osteoarthritis
OHS	Oxford Hip Score
PA	posterior approach
PATH	Percutaneously assisted total hip
PLA	posterolateral approach
PRISMA-ScR	Preferred Reporting Items for Systematic Reviews and Meta-Analyses Extension for Scoping Reviews
RCT	randomized controlled trial
ROM	range of motion
RoB	risk of bias
ROBINS-I	risk of bias in non-randomized studies of interventions
SF-36	Short Form 36
SuperCap	Supercapsular approach
SuperPATH	Supercapsular percutaneously assisted approach in total hip
THA	total hip arthroplasty
VAS	visual analog scale
WOMAC	Western Ontario and MacMaster Universities Osteoarthritis Index
